# Supplementary material for: Enhancing Tumor Immunity with IL-12 and PD-1 Blockade: A Strategy for Inducing Robust Central Memory T Cell Responses in Resistant Cancer Model
Source: Antibodies (Basel). 2024 Nov 20;13(4):94. doi: 10.3390/antib13040094 (PMC11586976; doi:10.3390/antib13040094)
Supplement: Supplementary file 1 [file antibodies-13-00094-s001.zip › antibodies-3078104-supplementary.pdf]

Supplementary Figure

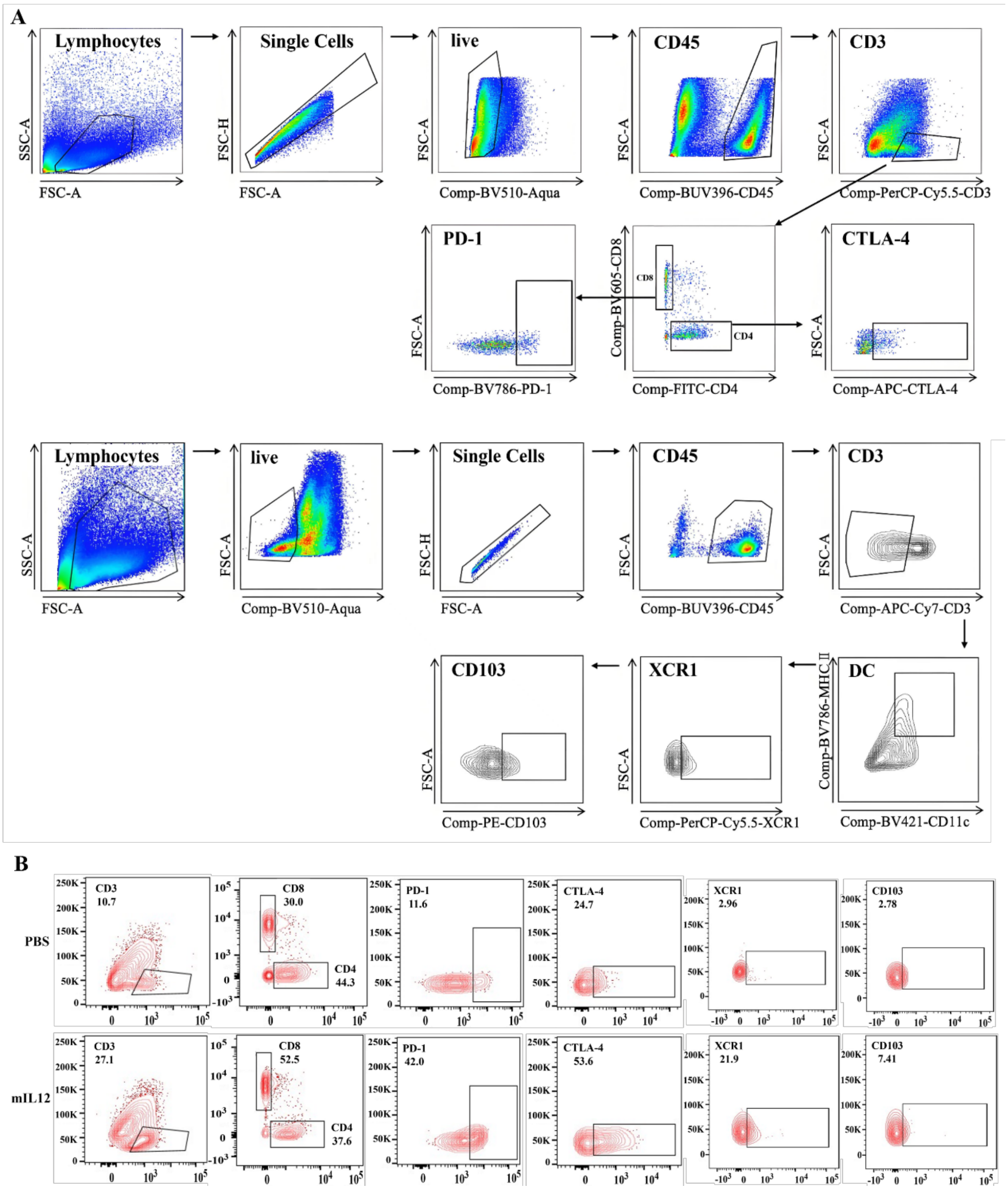

Figure S1. Analysis of T Cell and Dendritic Cell Populations in the MC38 Tumor Model. (A) Flow cytometry gating strategy was used to identify the following subsets: CD3$^+$, CD4$^+$, CD8$^+$, CTLA-4$^+$CD4$^+$, PD-1$^+$CD8$^+$, CD103$^+$DC$^+$, and XCR1$^+$DC$^+$. (B) Representative flow cytometry plots showcasing CD3$^+$, CD4$^+$, CD8$^+$, CTLA-4$^+$CD4$^+$, PD-1$^+$CD8$^+$, CD103$^+$DC$^+$ and XCR1$^+$DC$^+$ subsets.

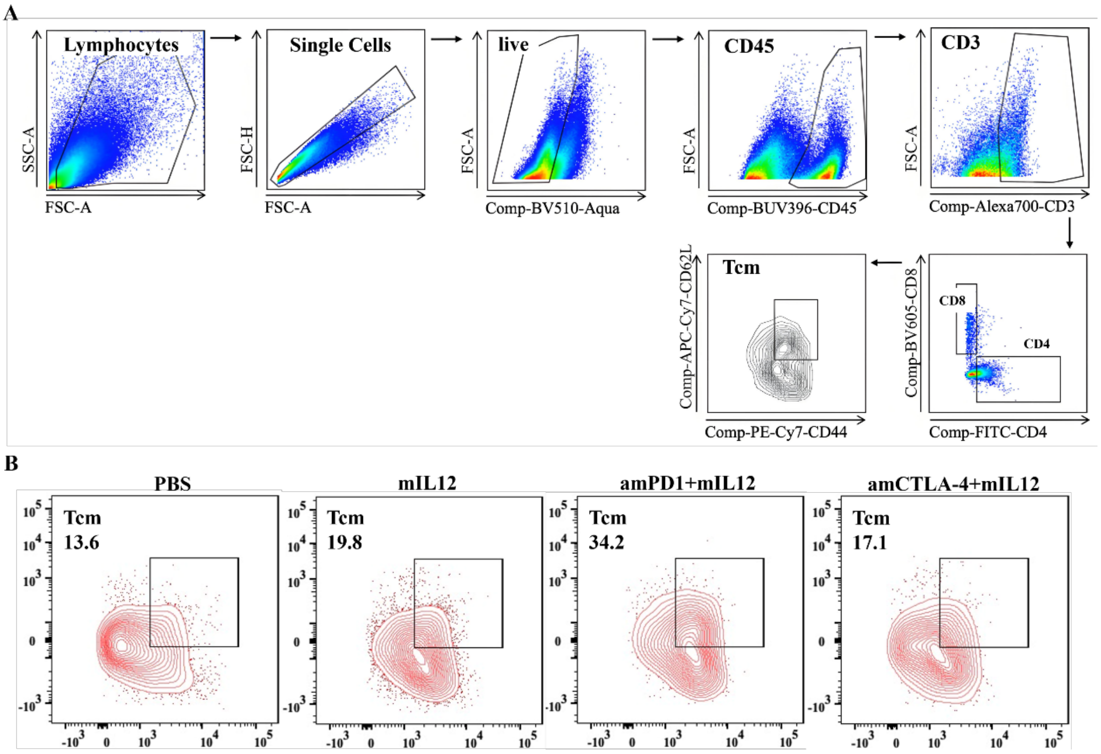

Figure S2. Analysis of Central Memory T Cells (Tcm) in the MC38 Tumor Model. (A) Flow cytometry gating strategy employed to identify Tcm subsets. (B) Representative flow cytometry plots illustrating Tcm subsets.
